# Supplementary material for: Multiple groups of neurons in the superior colliculus convert value signals into saccadic vigor
Source: bioRxiv. 2025 Jun 25:2025.06.24.661386. Preprint. [Version 1] doi: 10.1101/2025.06.24.661386 (PMC12262230; doi:10.1101/2025.06.24.661386)
Supplement: Supplement 1 — Supplementary Figure 1: Value modulation in representative SC neurons Example neurons recorded from SC. Raster plots (top) and spike density functions (bottom) aligned on target onset (left) and saccade onset (right) are shown. Red and blue plots showed trials when a saccade was directed to good vs. bad objects, respectively. Example neurons were selected to illustrate the different functional subtypes of SC neurons, namely: (A, B) Visual neuron, (C, D) Visuomotor neuron, (E, F) Motor neuron, and (G, H) Tonic neuron. Supplementary Figure-2: Schematic of the bootstrapping procedure. (A) The distribution of reaction time observed during the saccade task in each session is shown with trials grouped into three quantiles. (B) Firing rate histograms and raster plots of all recorded neurons are likewise divided into three groups according to behavioral quantile. (C) Simulated single-trial responses were generated by pooling 25 randomly selected responses from all recorded neurons. (D) 10–35 trials are simulated for each behavioral quantile. Population activity is generated by averaging over these simulated trials drawn from each behavioral quantile. (E) This bootstrapping is repeated 10,000 times to generate the averaged activity profiles, which show a clear effect of saccade RT on SC neurons. Supplementary Figure-3: Correlation between firing rate and saccade behvaior. A scatter plot between neuronal activity in the time window 80–160 and the saccade RT in the same trial is shown separately for a representative visual (A), visuomotor (B), motor (C), and tonic (D) neuron. The dashed line shows the best-fit line. The number on the top of each panel indicates Pearson’s correlation coefficient and the associated p-value. A scatter plot between neuronal activity in the time window 80–160 and the saccade PV in the same trial is shown separately for the same representative visual (E), visuomotor (F), motor (G), and tonic (H) neurons. The rest of the conventions are the same as a [file media-1.pdf]

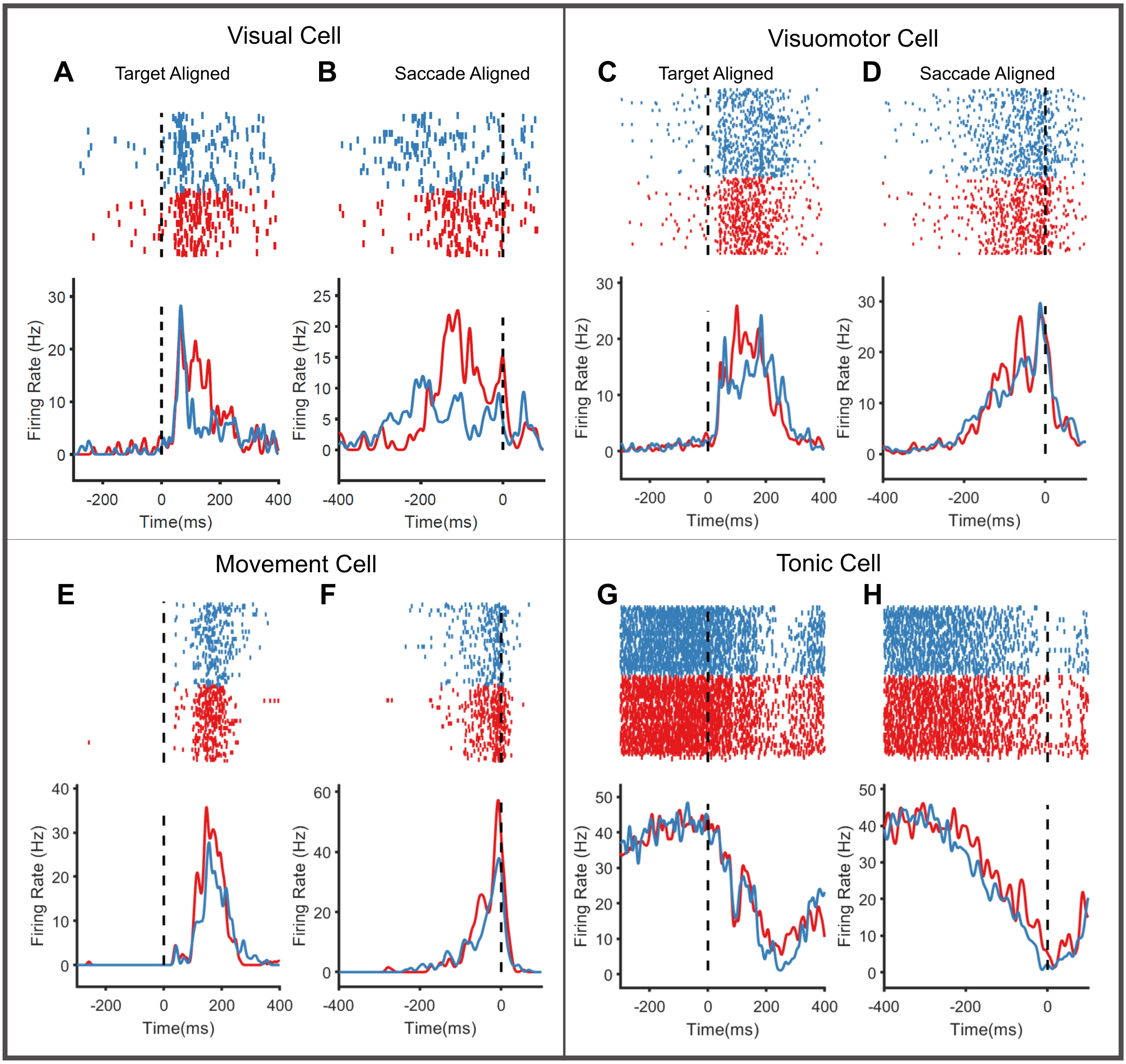

Supplementary Figure - 1

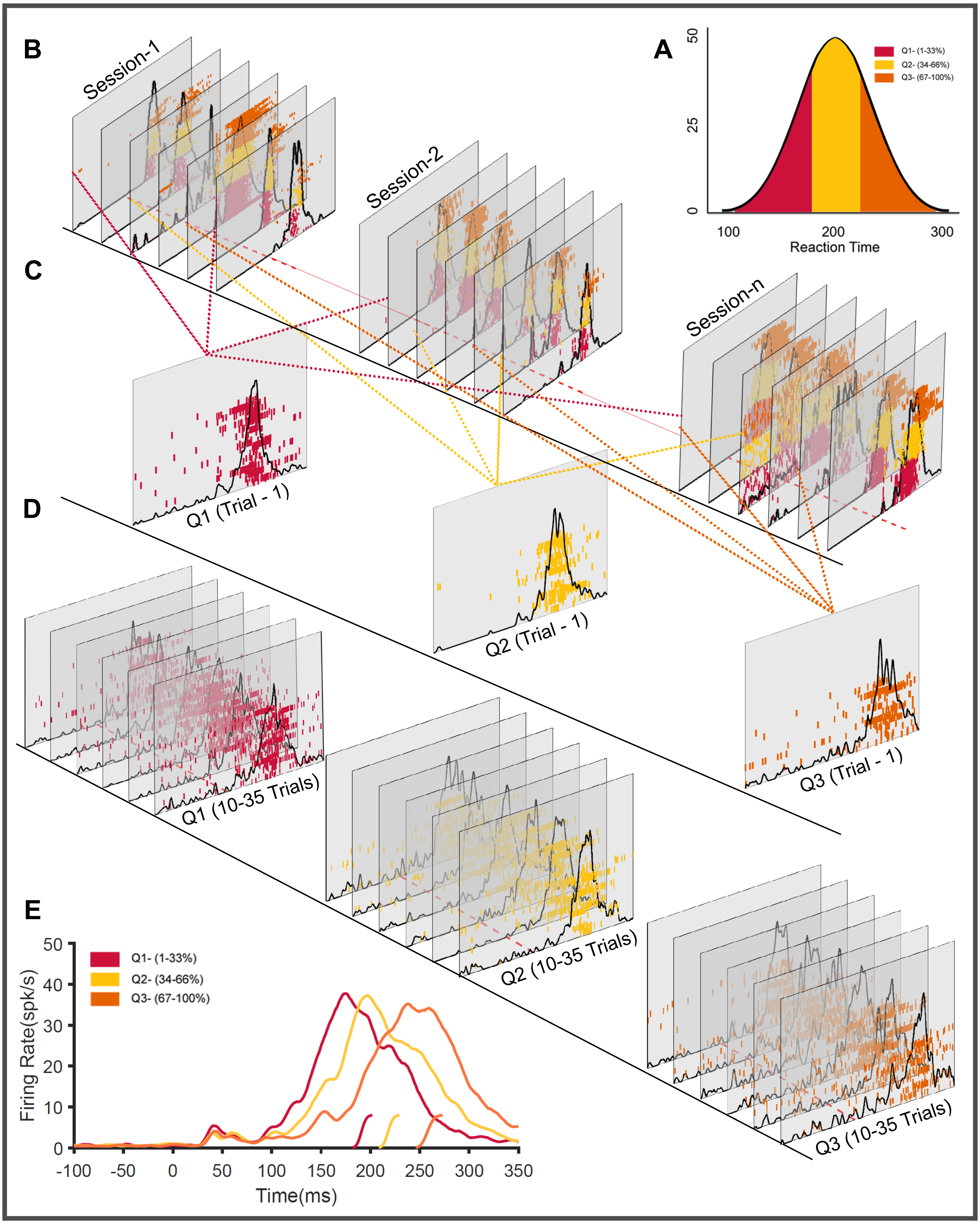

Supplementary Figure- 2

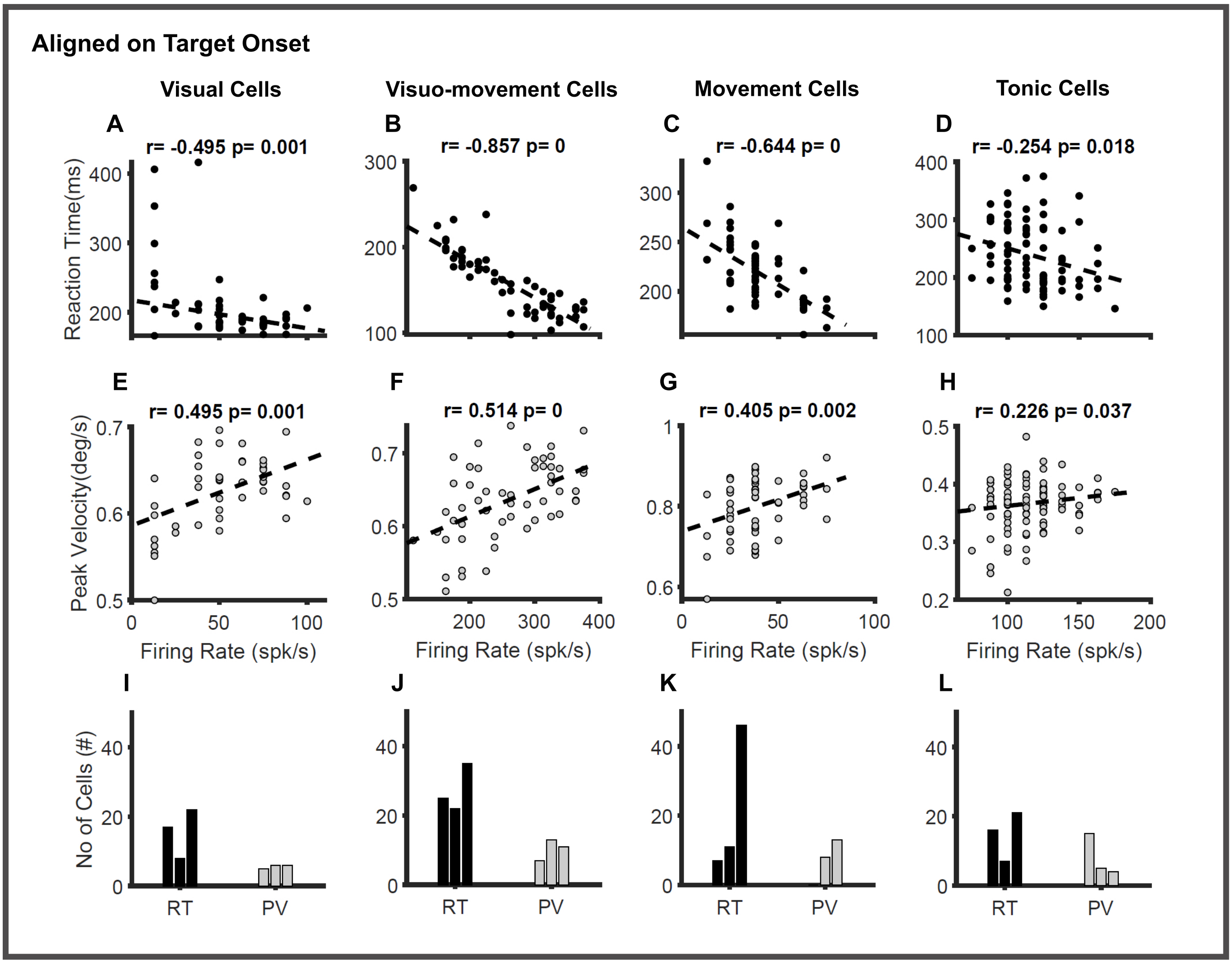

Supplementary Figure - 3

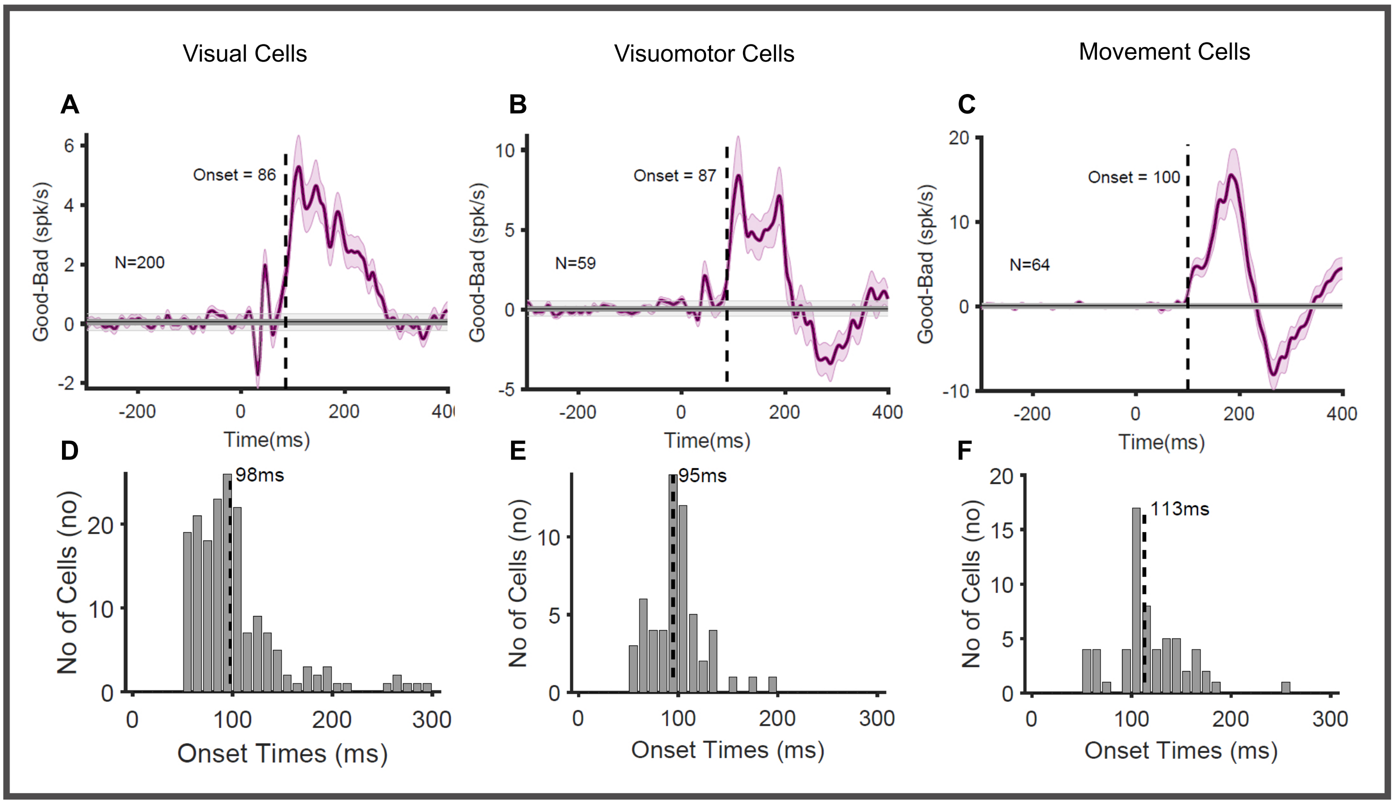

Supplementary Figure - 4

Table-1

Neuron Subtype	Epoch	Time to Peak (ms)								
		Short			Medium			Long		
		Mean	95% Intervals		Mean	95% Intervals		Mean	95% Intervals	
	E_{VIS}	**42**	39	45	**43**	40	47	**43**	40	47
**Visual Neuron**	L_{VIS}	**101**	97	105	**101**	98	105	**100**	96	105
	Pre_{SAC} (Target)	**164**	154	175	**166**	155	177	**184**	167	202
	Pre_{SAC} (Saccade)	**-86**	-100	-75	**-102**	-115	-87	**-130**	-148	-109
	E_{VIS}	**39**	38	41	**40**	39	42	**40**	39	41
**Visuomotor Neuron**	L_{VIS}	**109**	105	112	**110**	105	114	**109**	106	113
	Pre_{SAC} (Target)	**154**	147	164	**165**	157	174	**193**	183	201
	Pre_{SAC} (Saccade)	**-14**	-15	-11	**-13**	-16	-10	**-11**	-14	-8
	E_{VIS}	**44**	41	48	**45**	41	49	**44**	41	48
	L_{VIS}	**110**	102	118	**108**	102	113	**110**	105	114
**Motor Neuron**	Pre_{SAC} (Target)	**165**	158	171	**186**	180	191	**216**	207	225
	Pre_{SAC} (Saccade)	**-9**	-11	-8	**-8**	-9	-7	**-9**	-10	-8

Supplementary Table-1: Quantification of mean peak time in different phases of SC response.

A table showing the mean peak time for the simulated population activity for each reaction time quantile for visual, visuomotor, and motor neurons. The mean peak time and the 95% confidence intervals obtained by bootstrapping in each phase (E_{VIS}, L_{VIS}, and Pre_{SAC}) are separately tabulated. The values tabulated here are related to the analysis shown in Fig-5

Table- 2

Firing Rates (spk/s)													
**Subtype**	**Epoch**	**Short**				**Medium**				**Long**			
		**Mean**		**95% Interval**		**Mean**		**95% Interval**		**Mean**		**95% Interval**	
		Good	Bad	Good	Bad	Good	Bad	Good	Bad	Good	Bad	Good	Bad
**Visual Neuron**	E_{VIS}	31.37	28.51	29.6-33.3	26.7-30.4	30.32	28.24	28.6-32.2	26.5-30.1	27.29	26.51	25.5-29.1	24.8-28.3
	L_{VIS}	37.88	30.46	35.1-40.8	28.1-32.8	39.04	29.95	36.1-42.1	26.8-31.2	37.19	27.71	34.3-40.2	25.7-29.9
	Pre_{SAC}	29.18	23.65	26.6-31.8	21.5-25.9	30.76	23.08	28.3-33.4	21.2-25.1	30.00	20.07	27.5-32.7	18.33-21.9
**Visuomotor Neuron**	E_{VIS}	30.23	27.64	28.0-32.6	25.6-29.8	27.53	26.58	25.6-29.6	24.7-28.5	25.55	27.03	23.6-27.5	25.0-29.1
	L_{VIS}	51.21	43.62	47.5-55.0	40.4-46.9	46.02	35.10	42.7-49.4	32.3-37.9	38.60	33.66	35.6-41.7	31.0-36.3
	Pre_{SAC}	57.76	48.45	53.9-61.7	44.6-52.3	54.63	42.22	50.8-58.6	39.1-45.3	46.35	38.56	43.1-49.8	35.7-41.5
**Motor Neuron**	E_{VIS}	10.42	10.68	9.2-11.7	9.4-12.0	8.83	8.43	7.7-10.1	7.2-9.7	8.64	10.01	7.4-10.0	8.6-11.6
	L_{VIS}	34.86	29.40	32.1-37.7	26.8-32.0	27.26	21.50	24.8-29.8	19.4-23.7	20.60	19.49	18.6-22.7	17.5-21.6
	Pre_{SAC}	78.33	64.47	73.0-83.9	59.6-69.5	71.81	50.34	66.3-77.4	46.5-54.5	53.83	36.85	49.5-58.5	33.7-40.1

Supplementary Table-2: Quantification of value modulation in different phases of SC response.

A table showing the average firing activity of good and bad object trials during the E_{VIS} (40-80 ms), L_{VIS} (81-130ms), and Pre_{SAC} (131-170ms) phases of visual, visuomotor, and motor neurons. The mean firing rate and the 95% confidence interval obtained from the repeated bootstraps are tabulated separately for the three reaction time quantiles (short, medium, and long). The values tabulated here are related to the analysis shown in Fig-6
